# Current Insights in Cutaneous Lupus Erythematosus Immunopathogenesis

**DOI:** 10.3389/fimmu.2020.01353

**Published:** 2020-07-02

**Authors:** Colton J. Garelli, Maggi Ahmed Refat, Padma P. Nanaware, Zaida G. Ramirez-Ortiz, Mehdi Rashighi, Jillian M. Richmond

**Affiliations:** ^1^Department of Dermatology, University of Massachusetts Medical School, Worcester, MA, United States; ^2^Department of Pathology, University of Massachusetts Medical School, Worcester, MA, United States; ^3^Department of Medicine, University of Massachusetts Medical School, Worcester, MA, United States

**Keywords:** cutaneous, lupus, CLE, UV light, autoantibodies, interface dermatitis, lupus band, autoimmune

## Abstract

Cutaneous Lupus Erythematosus (CLE) is a clinically diverse group of autoimmune skin diseases with shared histological features of interface dermatitis and autoantibodies deposited at the dermal–epidermal junction. Various genetic and environmental triggers of CLE promote infiltration of T cells, B cells, neutrophils, antigen presenting cells, and NK cells into lesional skin. In this mini-review, we will discuss the clinical features of CLE, insights into CLE immunopathogenesis, and novel treatment approaches.

## Introduction

Cutaneous Lupus Erythematosus (CLE) is an autoimmune disease primarily affecting skin and mucosal tissue. Total CLE disease incidence is ~4.3 per 100,000 (1, 2). CLE exhibits a strong sex bias toward females much like systemic lupus erythematosus (SLE) (3–5). Certain CLE subtypes may progress to SLE (6, 7), which can have health consequences, including kidney and brain involvement leading to renal failure and neurologic disease (8). Successful treatment of cutaneous disease may significantly decrease the risk of systemic involvement (9). Treatment options for CLE are limited, with anti-malarials as the most commonly prescribed drugs, followed by calcineurin inhibitors, mycophenolate mofetil, methotrexate, and steroids (10–13).

## Clinical Features of CLE

CLE encompasses a heterogeneous group of photodermatoses with varying degrees of association with systemic disease (SLE) [reviewed in (14)]. It is typically classified into three main subtypes based on the disease chronicity, clinical morphology and distribution: acute (ACLE), subacute (SCLE), and chronic (CCLE) (15, 16). All CLE subtypes are characterized histopathologically by interface dermatitis (with the exception of tumid lupus and lupus panniculitis) and lupus band reaction, which consist of infiltration of immune cells and deposition of autoantibodies at the dermal-epidermal junction (DEJ) (17).

ACLE presents as transient erythematous patches that correspond to flares in SLE patients. A well-known example of ACLE is the malar rash, or butterfly rash, that classically crosses both cheeks but spares the nasolabial folds. This helps to distinguish it from other clinical mimickers such as rosacea and seborrheic dermatitis. ACLE can affect the entire body in some patients with bad flares, and is considered a criterion for the diagnosis of SLE. Up to 80% of SLE patients experience malar rash, which typically flares with UV exposure but does not leave any scar or dyspigmentation. Histologically, ACLE manifests as lymphoplasmacytic interface dermatitis with vacuolar changes at the DEJ associated with mucin deposition.

**Figure 1:**
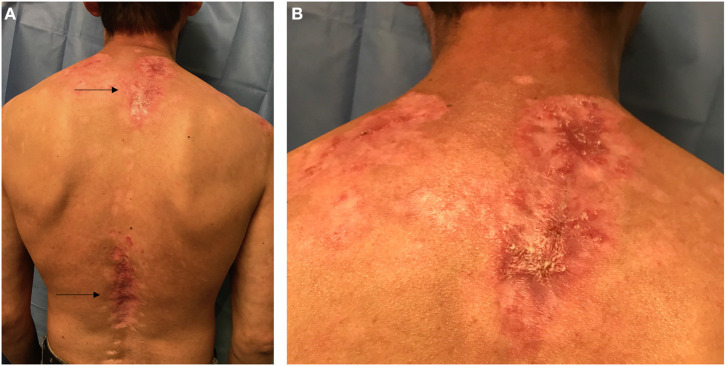
Posterior view of the trunk in a Hispanic patient with Cutaneous Lupus Erythematosus (CLE). **(A)** Arrows point to two lupus patches on the upper back and lower back. **(B)** Closer view of the upper back patch in the intrascapular area showing scaling, erythema, dyspigmentation, and scarring.

Lesions of SCLE last longer than the malar rash of ACLE, but systemic lupus occurs in a significantly lower percentage of the affected individuals. SCLE most often occurs on the photo-exposed areas of the upper chest, back, and external upper extremities (Figure 1). When active, it typically presents as papulosquamous lesions and/or annular plaques (psorasiform) with central clearing and raised erythematous scaly edges. Upon resolution, SCLE can leave dyspigmentation but no permanent scarring. SCLE is highly associated with anti-Ro/SSA autoantibodies. In patients with new-onset SCLE, it is important to carefully review medications, as SCLE can be induced by nonsteroidal anti-inflammatory drugs (NSAIDs), proton pump inhibitors, antihypertensives, and antifungals (18).

CCLE has multiple subtypes, and the most common is discoid LE (DLE). DLE commonly presents as red, atrophic, and hypopigmented plaques with a characteristic rim of hyperpigmentation. DLE typically only affects areas above the neck such as scalp, ears, nose, and cheeks (localized DLE). Presence of DLE is one of the diagnostic criteria of SLE and is observed in 20% of the patients with systemic disease; however, only 5–10% of cases with isolated DLE eventually progress to develop systemic disease (4). When it occurs on the scalp, DLE can cause irreversible scarring alopecia (19). Though rare, DLE may affect larger areas of the body including trunk and upper extremities (generalized DLE), which confers an increased risk of systemic involvement. DLE exhibits more prominent histopathological findings including follicular plugging, periadnexal lymphoplasmacytic infiltrate, and pigment incontinence. Other variants of CCLE, including tumid lupus, chilblain lupus, and lupus panniculitis, are significantly less frequent.

## Autoantibodies/Autoantigens

Like SLE, many CLE patients develop autoantibodies including anti-nuclear antibodies (ANAs). Antibodies against Ro/SSA and La/SSB are detected frequently and have been associated with SCLE and neonatal lupus erythematosus (NLE), in addition to SLE and Sjogren's Syndrome (20–24). Most CLE patients exhibit anti-Ro/SSA autoantibody reactivity patterns (25). Ro refers to ribonucleoproteins that are encoded by two separate gene products, resulting in 52 kDa (also known as TRIM21) and 60 kDa (also known as TROVE2) protein isotypes (26). CLE patients react more frequently to the 60 kDa form than the 52 kDa form (27). Anti-Ro autoantibodies may also be mechanistically involved in CLE pathogenesis, as commensal bacteria that produce a Ro60 kDa ortholog can trigger lupus development in mice (28). Infusion of anti-Ro/SSA into human skin grafted mice results in lupus band reaction similar to what is observed in CLE specimens (29).

Other autoantigens in CLE include SSB/La, ribonucleoprotein, smith (sm) antigen (30), C1q (31), and HMGB1 (32). UV damage can induce translocation of these autoantigens to the surface of keratinocytes, thereby making them bioavailable to the immune system (33–36). HMGB1 appears to play a significant role in the development of CLE: HMGB1 is highly expressed in the epidermis of CLE skin biopsies (37) and expression level correlates with clinically active photoinduced CLE lesions (38, 39).

A recent study in a cohort of Italian CLE patients found strong correlations between autoantibody specificities and CLE subtypes (40). CCLE is negatively associated with anti-extractable nuclear antigens (ENA), anti-Ro/SSA, and anti-dsDNA. SCLE positively correlates with ENA, anti-Ro/SSA, anti-Smith, and anti-RNP. ACLE is strongly associated with anti-dsDNA and ANA, though this may be due to the finding that these autoantibodies are found in higher frequencies in females and SLE patients.

NLE is a condition characterized by cutaneous, cardiac, and multi-systemic abnormalities observed in 5–16% of newborn infants whose mothers have autoantibodies against Ro/SSA, La/SSB, and U1-ribonucleoprotein (41–44), regardless of whether the mothers are symptomatic or not (45). Autoantibodies against Ro/SSA and La/SSB were detected in 98% of affected infants (45). Anti-Ro52/60-kDA Ro/SSA and 48-kD La/SSB auto-antibodies contribute to heart block (46), whereas 50-kD La/SSB are associated with cutaneous disease (47, 48), which is thought to self-resolve but can have long-term cutaneous changes (49). A study of 186 antibody exposed fetuses and infants indicates a direct correlation between the amount of maternal anti-Ro and anti-La antibodies and fetal tissue injury (48).

## CLE Immunopathogenesis

### Factors Contributing to Onset

Like many complex diseases, CLE is thought to arise from a combination of genetics and environment. Several immune genes have been implicated in subtypes of CLE, including cytokine genes, complement genes, and innate immune genes [reviewed in (50)]. Polymorphisms in the transcription factor IRF5, the signaling molecule TYK2, and the immune regulator CTLA4 were identified in a Finnish cohort of CLE patients (51). Familial chilblain lupus, a rare form of CLE characterized by acral lesions, is caused by gain-of-function mutations in the DNA sensor protein STING (52, 53), or mutations that decrease the exonuclease activity of TREX1 (54–56).

CLE and cutaneous involvement in SLE can be induced by UV radiation, and photosensitivity is a criterion used by the American College of Rheumatology for lupus diagnosis [reviewed in (57, 58)]. The pathogenic wavelengths of UV radiation remain unclear. However, UVB is considered an instigating factor, as the dosage required to induce erythema is 1,000 fold less than that of UVA in lupus patients (59). Lupus keratinocytes are more sensitive to UV light than healthy keratinocytes (60), and exhibit aberrant apoptosis thereby generating cellular debris and activating the immune system (57). Some groups have reported impaired clearance and accumulation of apoptotic keratinocyte debris in afflicted skin (61), while others have found inflammatory clearance with no evidence of impaired clearance (62). Regardless of clearance efficacy, it is clear that UV damaged, apoptotic keratinocytes are one of the main instigating factors in CLE lesion formation. A recent review by Wolf et al. (63) summarized known aspects of UV-induced CLE.

Over 100 drugs have been identified that induce CLE or CLE flares (18, 64–66), with SCLE being the most common clinical subtype induced by prescribed medications such as antihypertensives and antifungals (67). The blood pressure medication hydrochlorothorazide induces SCLE in a photosensitive manner (68). The chemotherapeutic 5-fluorouracil can cause SCLE or DLE (69, 70). Proton pump inhibitors such as omeprazole are triggers of SCLE (71, 72). There are a few case reports of CLE resulting after anti-TNF therapy, though the etiology is not fully understood (73).

Smoking is a trigger of CLE (74), particularly DLE (75), and reduces responsiveness to anti-malarial treatments (76, 77). Counseling patients to quit smoking can significantly improve CLE and other autoimmune conditions (77–80). Case reports demonstrate environmental or occupational exposures can also induce CLE, such as silica exposure (81, 82). Lastly, microbes may trigger CLE: recent studies found *Staphylococcus aureus* can colonize skin following IFN-mediated barrier disruption (83), and is enriched in CLE lesional skin (83).

### Sensing Damage: Innate Immune Cells

UVB radiation and/or drugs cause keratinocyte damage and death, which is sensed by the innate immune system to create a feed-forward loop driving pathogenesis. Keratinocytes themselves can respond to TLR-independent nucleic acid ligands via MDA5, RIG-I, c-GAS and STING, and can activate the AIM2 inflammasome, which can initiate the interferon response [reviewed in (84)]. Thus, they can respond to bystander damage to alert the immune system.

**Figure 2:**
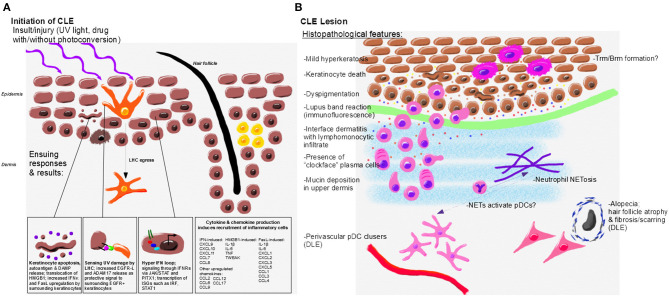
Initiation, immunopathogenesis, and histopathologic features of CLE. **(A)** Environmental triggers of CLE include UV light and drugs which may become photoconverted to induce translocation of autoantigens, DAMP release and keratinocyte death. UV damage is sensed by LHC, which protect surrounding keratinocytes through release of EGFR-L; however, LHC are decreased or absent in CLE lesional skin. Keratinocytes from CLE patients also exhibit a hyper IFN loop via JAK/STAT signaling to release IFNs. Keratinocytes and skin resident immune cells upregulate cytokines and chemokines in response to IFN and other signals to promote lesion formation. **(B)** Histopathological features of CLE lesions include mild hyperkeratosis, keratinocyte death and debris, lymphocytic infiltrates, interface dermatitis, and mucin deposition. Dyspigmentation occurs in some CLE subtypes. Perivascular pDC clusters and fibrosing alopecia occur primarily in DLE lesions. CLE interface dermatitis is comprised of lymphomonocytic infiltrates with “clockface” plasma cells. Neutrophils, which may form NETs, are also present in CLE lesions and may activate pDCs through release of DNA and other autoantigens. Resident memory T and B cells likely form in CLE lesions. Inflammatory mediators drive continued recruitment and damage in the lesion, including IFNκ (yellow, associated with keratinocytes), IFNγ (orange, associated with the infiltrating immune cells), CXCL9 (red, amplified by keratinocytes and immune cells), and CXCL10 (blue, amplified by keratinocytes and immune cells).

Langerhans cells are specialized dendritic cell (DC) populations that live in the epidermis, and migrate to skin draining lymph nodes upon antigen encounter (85). UVB-induced keratinocyte damage is sensed by Langerhans cells in both lupus-prone [MRL/lpr and B6.SLE1yaa (86)] and wild type mice (87). Shipman et al. (86) demonstrated that ADAM17 is upregulated in Langerhans cells following UVB exposure, which in turn increases conversion of EGFR ligands into an active form in an attempt to protect keratinocytes from further UVB damage. In murine SLE models, Langerhans cells have a reduced ability to process EGFR ligands into an active form, resulting in a dysfunctional LC-KC axis in CLE lesions. LCs are subsequently replaced by other inflammatory DC subsets, thereby promoting further inflammation (88, 89) (Figure 2A). These data are supported by a recent microarray study of CLE biopsies that demonstrated decreased EGFR signaling pathways (90).

One particularly important inflammatory DC subset contributing to CLE pathogenesis is plasmacytoid DCs (pDCs). pDCs are common in DLE, and are used by dermatopathologists to assist in diagnosis (91). When presented with DNA, pDCs potently upregulate Type-1 IFN, mainly IFNα. pDCs are a key source of Type-1 IFN in lupus lesional skin (92, 93), and UVB promotes their recruitment to the skin (94). A first-in-human study of BDCA2 antibody (BIIB059), which targets pDCs, for SLE decreased expression of IFN response genes in blood, normalized MxA expression, reduced immune infiltrates in skin lesions, and decreased CLASI-A score [(95), NCT02847598]. BIIB059 is now in a phase-2 clinical trial for SLE and active CLE treatment (NCT02847598).

In addition to apoptotic keratinocytes, another potential source of DNA that could activate pDCs in the skin is from neutrophils. Some neutrophils have the ability to produce neutrophil extracellular traps (NETs), which are comprised of DNA, chromatin and various proteins. NETs have been found in various CLE subtypes including: lupus panniculitis, ACLE, DLE, and to a lesser extent, SCLE (96). Though the study by Safi et al. (96) included a cohort of only 30 patients, their work indicates the presence and contribution of NETs in CLE is worth further investigation. A subclass of neutrophils, called low density granulocytes (LDGs), have an increased propensity for producing NETs. LDGs have been reported in the skin of SLE patients (97). LDGs NETs provide a source of autoantigens, and may interact with nucleic acids from UV-B damaged KCs. Subsequent accumulation of apoptotic DNA provides a potential mechanism by which skin lesions are initiated or sustained (98) (Figure 2B).

Like DC populations, macrophages and monocytes are also involved in debris clearance and sensing of DAMPs. Immunohistochemical studies demonstrated that CD68^+^ macrophages express FasL and are densely populated near hair follicles in CLE lesions (99). Inflammasome activity in blood monocytes from SLE patients is enhanced via type I IFN-mediated upregulation of IRF1 (100), though the functional capacity of macrophages in CLE has not been well-studied. A trial of macrophage colony-stimulating factor (MCSF) antibody failed to reduce immune infiltrates or activation in CLE lesions and did not improve CLASI score (101). Thus, it is possible that tissue macrophages in CLE lesions perform an immune regulatory function, require different cytokines for their function/survival, or are dispensable for CLE.

Another aspect of innate immune involvement in lupus is the uptake and processing of cellular debris for both clearance and presentation of autoantigens. The scavenger receptor C1q binds to keratinocyte apoptotic blebs to assist their clearance (102). A silent single nucleotide polymorphism (SNP) in C1QA gene (Gly70_GGG/GGA_) results in lower serum C1q and is associated with SCLE (103).

### Promoting Damage: Lymphocytic Infiltrates

Inflammatory infiltrates in CLE are comprised mainly of T lymphocytes, with other infiltrating cells including B cells/plasma cells, NK cells, dendritic cells (104, 105), and in some subtypes, neutrophils (106), implicating these populations as key drivers of inflammation in CLE lesions. The T cell specificities in CLE skin are unknown, but SLE studies identified T cells reactive to nucleosomes/histones (107), which can induce anti-dsDNA antibody production (108). TCR-Vβ38 and Vβ13 were enriched in skin of CCLE patients, implicating oligoclonal expansion (109). Several studies have observed expression of cytotoxic markers characteristic of T cell function, such as Th1-related cytokines and granzyme B (110, 111). Interestingly, the perforin promoter (112), CD70 promoter (113), and other loci (114) are hypomethylated in CD4^+^ T cells from SCLE patients, making them poised to transcribe effector molecules. Both CD4^+^ and CD8^+^ circulating T cells have higher HLA-DR expression in CLE patients, and higher CD25 in DLE patients specifically, compared to healthy controls (115). Tregs are lower (116, 117), and γδ T cells are higher (118), in CLE lesions. Skin-infiltrating CD4^+^ T cells express FasL have the potential to ligate Fas and can induce apoptosis in keratinocytes and other infiltrating immune cells (99).

Plasma cells in lupus skin lesions are often observed as “clockface” cells (119). It is not clear whether lupus band reactions arise from local production of autoantibodies in the skin by plasma cells, or if they deposit in the skin from the circulation. Interestingly, while B cells are required for development of lupus in mice (120, 121), autoantibody formation appears to be a byproduct of lupus immune responses and not a driving factor: MRL-lpr mice with B cells incapable of secreting immunoglobulin develop nephritis, implicating B cells' antigen presenting function as being critical for lupus development (122). It is unclear which B cell functions are required for CLE, though preclinical studies using chimeric antigen receptor T cells (CAR-Ts) directed against B cells ameliorated skin disease in lupus-prone mice (123). Furthermore, B cell depletion with rituximab showed efficacy in a case study of 4 lupus panniculitis patients with childhood onset who were refractory to other standard treatments (124), and ameliorated skin symptoms in a retrospective case study of 14 consecutive SLE, one CCLE and two SCLE patients with recalcitrant skin involvement (125).

The atypical lymphocyte marker CD38 (ADP-ribosyl cyclase/cyclic ADP-ribose hydrolase) is highly expressed in CLE lesional skin. CD38 was recently shown to be important for Tfh-B cell collaboration in response to recurrent influenza vaccination (126). Polymorphisms in intron 1 of CD38 are associated with the development of DLE in a Spanish patient population (127). It is unclear what role CD38 plays in CLE pathogenesis, though knocking CD38 out of MRL-lpr mice accelerates lupus (128).

NK cells have been studied in peripheral blood from SLE patients, though their precise roles in CLE and skin are not known due to a paucity of mechanistic studies. In the SLE studies, blood NK numbers decrease with increased lupus disease activity and/or exhibit defects in traditional killing functions (129–131); though rare populations are often expanded and secrete higher levels of IFNγ compared to healthy controls (132–135). Given their roles in sensing cellular stress (136), clearing tumors (137, 138), and keratinocyte-derived tumors in particular (139), it is likely that NK cells are also able to kill stressed keratinocytes following UV or drug injury to promote CLE lesions. It is also possible that T cells expressing killer receptors contribute to keratinocyte death in CLE, as exemplified by NKG2D ligation on mouse dendritic epidermal T cells (DETCs): in the absence of TCR signaling, NKG2D ligation on DETCs induces IFNγ production and causes keratinocyte cytotoxicity (140). In line with this, one study found invariant NK T cells (iNKTs) were enriched in SCLE and DLE patient blood by flow cytometry and in lesional skin by immunohistochemistry (141): they expressed IFNγ *in situ*, and were Ki67+, indicating they were proliferating in skin lesions.

### Chemokine Recruitment of Leukocytes to CLE Lesions

Type 1 IFN and type 2 IFN signaling stimulates expression of CXCL9, CXCL10, and CXCL11 which mediate leukocyte migration to peripheral tissue via CXCR3. IFN and CXCR3 have been postulated to drive the pathogenesis of all subtypes CLE (142), mediating the recruitment of the aforementioned immune cell types and providing positive feedback loops for T cell and pDC recruitment. Previous studies reported CXCR3 ligand expression in the skin in all the different subtypes of CLE (57, 58, 104, 143). Further, UV light induces upregulation of CXCR3 ligands in keratinocytes, linking the environmental insult to the recruitment of pathogenic immune cells (58).

Type 1 IFN also induces CXCL13 (144), which can support germinal center formation through migration of Tfh cells and B cells [reviewed in (145)]. While CXCL13 serves as a biomarker of SLE but not CLE (146), epidermal injury can accelerate nephritis in NZM2328 mice via upregulation of CXCL13 (147). The receptor for CXCL13, CXCR5, can influence B cell function by enhancing antigen uptake via membrane ruffling and LFA-1-mediated adhesion, and integrating BCR signaling in motile cells (148). The precise role of CXCL13 in CLE is not known.

CCL17 is expressed by keratinocytes in CLE lesions and has been hypothesized to recruit CD8^+^ T cells bearing the cognate receptor CCR4 to the skin in scarring CLE (149). iNKT cells in CLE lesions also express CCR4, and blood iNKTs express higher levels of CCR4 and CCR6 than healthy controls (141). Higher CCR5 and lower CCR3 expression on peripheral CD4+ T cells is associated with higher disease activity in CLE (150).

Gene expression analysis of human DLE and SCLE skin biopsies, as well as a mouse model of CLE, exhibited increased CCL3, CCL4, CCL7, and CCL8 (151). While the precise roles of these chemokines in CLE have yet to be elucidated, inferences can be made based on their previously established biological activities. Future studies will need to be conducted to determine how these chemokines guide specific immune populations to and through the skin during CLE.

### Paracrine Signals: Cytokines, Hormones, and Master Regulators

The inflammatory signatures of CLE are interferon (IFN)-based: a recent microarray study of 90 CLE biopsies found increased IFN pathways in all CLE subtypes, and DLE samples had a unique IFNγ node (90). Interestingly, the authors found no differences between skin biopsies of patients with and without systemic involvement. Blocking IFNα receptor improved CLASI scores for SLE patients with cutaneous involvement in a phase II trial [(152, 153), reviewed in (154)]; however IFNγ blockade did not improve CLASI scores in DLE patients (155). Single cell resolution may be necessary to elucidate the precise roles of different IFNs in pathogenesis.

Keratinocytes from CLE patients exhibit an enhanced response to both type-1 and type-2 IFNs (156), and produce IFNκ (157), IL-6 (158), and Type-III IFN (IFNλ) following UVB damage. Cytokine dysregulation in UVB treated CLE keratinocytes provides a link between UV initiation factors and immunopathogenesis (156). UV treatment of keratinocytes induces upregulation of IFNκ, which plays a key homeostatic role in maintaining IFN balance in skin (158). Lupus keratinocytes derived from active CLE lesions and nonlesional skin constitutively overexpress IFNκ, which increases photosensitivity via plasmacytoid DC production of IFNα in response to IFNκ signaling (157).

Higher levels of Th1 and Th17 cells in CLE lesions have been reported, along with IL-21 (159). IL-21 activates pDCs to produce granzyme B (160), thereby enhancing keratinocyte killing by NK cells. Interestingly, Salvi et al. (159) found that type I IFN served as a negative regulatory loop for granzyme B production by pDCs. This implies that high levels of type I IFN in skin are not pathogenic, but rather represent an attempt to counter inflammation.

The contribution of female sex hormones in CLE remains unclear. One epidemiological study noted premenstrual and perimenopausal flares (161), indicating that a certain level of estrogen is protective. Nevertheless, the addition of estrogen to keratinocyte cultures doubles Ro/SSA surface expression following UVB exposure (162) and enhances binding of anti-Ro/SSA and La/SSB autoantibodies to the plasma membrane (163). A patient who received estrogen as part of sex reassignment surgery developed tumid lupus following UV exposure (164). Estrogen can positively regulate the IFNγ promoter (165) and NK cell activity (166), possibly explaining other roles in CLE pathogenesis.

Vgll3 is a transcription co-factor that governs expression of inflammatory genes associated with autoimmunity, including CLE (167). Vgll3 is more highly expressed in female skin and in lupus patient skin regardless of gender. Overexpression of Vgll3 in male mice makes skin appear more “female-like” and promotes both skin and systemic autoimmune attack (168).

## Conclusion

The complexity of CLE has made it difficult to fully elucidate pathogenesis, with genetic and environmental triggers causing both innate and adaptive immune activation that creates diverse clinical manifestations. Nevertheless, increased knowledge in this field has paved the wave for promising new drugs in CLE. Blocking IFNAR antibody (Anifrolumab) improved CLASI scores in a phase IIb double-blind trial (>50% improvement, *p* = 0.013) (152, 153). Rituximab B cell depletion showed efficacy in case reports of CLE subtypes and SLE with skin involvement (124). The JAK/STAT pathways mediate signaling for a myriad of cytokines, including IFN, and other biological processes. JAK inhibition with tofacitinib (JAK3>JAK1>JAK2), baricitinib (JAK1/2) or ruxolitinib (JAK1/2) showed efficacy for familial chilblain lupus in human case studies and small clinical trials (53, 169–171). However, filgotinib (JAK1) did not reach its primary endpoint in a phase II double blind study, underscoring the need for a better understanding of which JAKs and STATs drive CLE (172–174). Similarly, the roles of IL-12 and IL-23 need to be revisited, as ustekinumab has been reported to both treat (175–178) and cause CLE (179).

Another approach to treat CLE is to enhance Treg function. The immune-dampening peptide Edratide, which stimulates Tregs, was safe in a phase II trial for SLE and improved BILAG score, but did not meet its primary endpoint (180). CAR-Tregs, which have been shown to be efficacious in a mouse model of asthma (181), may provide another approach, though they have yet to be tested for lupus (182). Of note, a challenge that remains in all CLE and lupus trials is the fact that patients are maintained on immunosuppressive drugs to keep their autoimmunity in check, which may have the unintended consequence of masking true efficacy. Ultimately, novel immunotherapies will need to be tested and developed for treatment of all CLE subtypes.

## Ethics Statement

Written informed consent was obtained from the individual for the publication of any potentially identifiable images or data included in this article.

## Author Contributions

MAR: patient photos and consent and figure generation. JR: artwork/figure generation. MR: clinical section. ZR-O: innate immune genes/receptors of cellular debris section. PN: autoantibodies/autoantigens section. CG and JR: all other sections. All authors approved the final manuscript. All authors contributed to the article and approved the submitted version.

## Conflict of Interest

JR is an inventor on patent application #62489191, “Diagnosis and Treatment of Vitiligo” which covers targeting IL-15 and Trm for the treatment of vitiligo; and on patent application #15/851,651, “Anti-human CXCR3 antibodies for the Treatment of Vitiligo” which covers targeting CXCR3 for the treatment of vitiligo. JR is a reviewer for Frontiers Immunology journal. The remaining authors declare that the research was conducted in the absence of any commercial or financial relationships that could be construed as a potential conflict of interest.

## References

[1] Hejazi EZ Werth VP. Cutaneous lupus erythematosus: an update on pathogenesis, diagnosis and treatment. Am J Clin Dermatol. (2016) 17:135–46. 10.1007/s40257-016-0173-9 26872954

[2] Durosaro O Davis MDP Reed KB Rohlinger AL. Incidence of cutaneous lupus erythematosus, 1965-2005: a population-based study. Arch Dermatol. (2009) 145:249–53. 10.1001/archdermatol.2009.21 19289752 PMC3953616

[3] Lockshin MD Mary Kirkland Center for Lupus Research Consortium. Biology of the sex and age distribution of systemic lupus erythematosus. Arthritis Rheum. (2007) 57:608–11. 10.1002/art.22676 17471529

[4] Merola JF Prystowsky SD Iversen C Gomez-Puerta JA Norton T Tsao P. Association of discoid lupus erythematosus with other clinical manifestations among patients with systemic lupus erythematosus. J Am Acad Dermatol. (2013) 69:19–24. 10.1016/j.jaad.2013.02.010 23541758 PMC3686921

[5] Wahie S Daly AK Cordell HJ Goodfield MJ Jones SK Lovell CR. Clinical and pharmacogenetic influences on response to hydroxychloroquine in discoid lupus erythematosus: a retrospective cohort study. J Invest Dermatol. (2011) 131:1981–6. 10.1038/jid.2011.167 21734714

[6] Chong BF. Understanding how cutaneous lupus erythematosus progresses to systemic lupus erythematosus. JAMA Dermatol. (2014) 150:296. 10.1001/jamadermatol.2013.9030 24477964

[7] Kirchhof MG Dutz JP. The immunopathology of cutaneous lupus erythematosus. Rheum Dis Clin North Am. (2014) 40:455–74. 10.1016/j.rdc.2014.04.006 25034156

[8] Yu C Gershwin ME Chang C. Diagnostic criteria for systemic lupus erythematosus: a critical review. J Autoimmun. (2014) 48–9:10–3. 10.1016/j.jaut.2014.01.004 24461385

[9] Stannard JN Kahlenberg JM. Cutaneous lupus erythematosus: updates on pathogenesis and associations with systemic lupus. Curr Opin Rheumatol. (2016) 28:453–9. 10.1097/BOR.0000000000000308 27270345 PMC4965280

[10] Schultz HY Dutz JP Furukawa F Goodfield MJ Kuhn A Lee LA. From pathogenesis, epidemiology, and genetics to definitions, diagnosis, and treatments of cutaneous lupus erythematosus and dermatomyositis: a report from the 3rd International Conference on Cutaneous Lupus Erythematosus (ICCLE) 2013. J Invest Dermatol. (2015) 135:7–12. 10.1038/jid.2014.316 25501376 PMC4921244

[11] Reich A Werth VP Furukawa F Kuhn A Szczech J Samotij D. Treatment of cutaneous lupus erythematosus: current practice variations. Lupus. (2016) 25:964–72. 10.1177/0961203316628997 26821963

[12] Chang AY Werth VP. Treatment of cutaneous lupus. Curr Rheumatol Rep. (2011) 13:300–7. 10.1007/s11926-011-0180-z 21503694 PMC3245840

[13] Kuhn A Ruland V Bonsmann G. Cutaneous lupus erythematosus: update of therapeutic options part II. J Am Acad Dermatol. (2011) 65:e195–213. 10.1016/j.jaad.2010.06.017 20739095

[14] Werth VP. Clinical manifestations of cutaneous lupus erythematosus. Autoimmun Rev. (2005) 4:296–302. 10.1016/j.autrev.2005.01.003 15990077

[15] Obermoser G Sontheimer RD Zelger B. Overview of common, rare and atypical manifestations of cutaneous lupus erythematosus and histopathological correlates. Lupus. (2010) 19:1050–70. 10.1177/0961203310370048 20693199

[16] Okon LG Werth VP. Cutaneous lupus erythematosus: diagnosis and treatment. Best Pract Res Clin Rheumatol. (2013) 27:391–404. 10.1016/j.berh.2013.07.008 24238695 PMC3927537

[17] Bennion SD Middleton MH David-Bajar KM Brice S Norris DA. In three types of interface dermatitis, different patterns of expression of intercellular adhesion molecule-1 (ICAM-1) indicate different triggers of disease. J Invest Dermatol. (1995) 105:71S−9S. 10.1111/1523-1747.ep12316107 7616001

[18] Michaelis TC Sontheimer RD Lowe GC. An update in drug-induced subacute cutaneous lupus erythematosus. Dermatol Online J. (2017) 23:13030/qt55x42822. 28329511

[19] Wilson CL Burge SM Dean D Dawber RP. Scarring alopecia in discoid lupus erythematosus. Br J Dermatol. (1992) 126:307–14. 10.1111/j.1365-2133.1992.tb00670.x 1373948

[20] Chan EK Andrade LE. Antinuclear antibodies in Sjögren's syndrome. Rheum Dis Clin North Am. (1992) 18:551–70. 1496161

[21] García-Carrasco M Fuentes-Alexandro S Escárcega RO Salgado G Riebeling C Cervera R. Pathophysiology of Sjögren's syndrome. Arch Med Res. (2006) 37:921–32. 10.1016/j.arcmed.2006.08.002 17045106

[22] Kobayashi R Mii S Nakano T Harada H Eto H. Neonatal lupus erythematosus in Japan: a review of the literature. Autoimmun Rev. (2009) 8:462–6. 10.1016/j.autrev.2008.12.013 19162245

[23] Sontheimer RD Maddison PJ Reichlin M Jordon RE Stastny P Gilliam JN. Serologic and HLA associations in subacute cutaneous lupus erythematosus, a clinical subset of lupus erythematosus. Ann Intern Med. (1982) 97:664–71. 10.7326/0003-4819-97-5-664 6982644

[24] Wasicek CA Reichlin M. Clinical and serological differences between systemic lupus erythematosus patients with antibodies to Ro versus patients with antibodies to Ro and La. J Clin Invest. (1982) 69:835–43. 10.1172/JCI110523 6804494 PMC370138

[25] Patsinakidis N Gambichler T Lahner N Moellenhoff K Kreuter A. Cutaneous characteristics and association with antinuclear antibodies in 402 patients with different subtypes of lupus erythematosus. J Eur Acad Dermatol Venereol. (2016) 30:2097–104. 10.1111/jdv.13769 27431977

[26] Itoh K Itoh Y Frank MB. Protein heterogeneity in the human Ro/SSA ribonucleoproteins. The 52- and 60-kD Ro/SSA autoantigens are encoded by separate genes. J Clin Invest. (1991) 87:177–86. 10.1172/JCI114968 1985094 PMC295020

[27] Popovic K Brauner S Ek M Wahren-Herlenius M Nyberg F. Fine specificity of the Ro/SSA autoantibody response in relation to serological and clinical findings in 96 patients with self-reported cutaneous symptoms induced by the sun. Lupus. (2007) 16:10–7. 10.1177/0961203306073135 17283579

[28] Greiling TM Dehner C Chen X Hughes K Iñiguez AJ Boccitto M. Commensal orthologs of the human autoantigen Ro60 as triggers of autoimmunity in lupus. Sci Transl Med. (2018) 10:eaan2306. 10.1126/scitranslmed.aan2306 29593104 PMC5918293

[29] Lee LA Gaither KK Coulter SN Norris DA Harley JB. Pattern of cutaneous immunoglobulin G deposition in subacute cutaneous lupus erythematosus is reproduced by infusing purified anti-Ro (SSA) autoantibodies into human skin-grafted mice. J Clin Invest. (1989) 83:1556–62. 10.1172/JCI114052 2651477 PMC303861

[30] Vera-Recabarren MA García-Carrasco M Ramos-Casals M Herrero C. Cutaneous lupus erythematosus: clinical and immunological study of 308 patients stratified by gender. Clin Exp Dermatol. (2010) 35:729–35. 10.1111/j.1365-2230.2009.03764.x 20015282

[31] Bălănescu E Tănăsescu C Bălănescu P Olteanu R Badea C Petrutescu B. Anti C1q antibodies in cutaneous lupus erythematosus. Rom J Intern Med. (2010) 48:159–63. 21428180

[32] Hayashi A Nagafuchi H Ito I Hirota K Yoshida M Ozaki S. Lupus antibodies to the HMGB1 chromosomal protein: epitope mapping and association with disease activity. Mod Rheumatol. (2009) 19:283–92. 10.3109/s10165-009-0151-7 19214652

[33] Lawley W Doherty A Denniss S Chauhan D Pruijn G van Venrooij WJ. Rapid lupus autoantigen relocalization and reactive oxygen species accumulation following ultraviolet irradiation of human keratinocytes. Rheumatology. (2000) 39:253–61. 10.1093/rheumatology/39.3.253 10788532

[34] Caricchio R McPhie L Cohen PL. Ultraviolet B radiation-induced cell death: critical role of ultraviolet dose in inflammation and lupus autoantigen redistribution. J Immunol. (2003) 171:5778–86. 10.4049/jimmunol.171.11.5778 14634086

[35] Abdulahad DA Westra J Limburg PC Kallenberg CGM Bijl M. HMGB1 in systemic lupus erythematosus: its role in cutaneous lesions development. Autoimmun Rev. (2010) 9:661–5. 10.1016/j.autrev.2010.05.015 20546955

[36] Abdulahad DA Westra J Reefman E Zuidersma E Bijzet J Limburg PC. High mobility group box1 (HMGB1) in relation to cutaneous inflammation in systemic lupus erythematosus (SLE). Lupus. (2013) 22:597–606. 10.1177/0961203313483377 23549344

[37] Popovic K Ek M Espinosa A Padyukov L Harris HE Wahren-Herlenius M. Increased expression of the novel proinflammatory cytokine high mobility group box chromosomal protein 1 in skin lesions of patients with lupus erythematosus. Arthritis Rheum. (2005) 52:3639–45. 10.1002/art.21398 16255056

[38] Garcia-Romo GS Caielli S Vega B Connolly J Allantaz F Xu Z. Netting neutrophils are major inducers of type I IFN production in pediatric systemic lupus erythematosus. Sci Transl Med. (2011) 3:73ra20. 10.1126/scitranslmed.3001201 21389264 PMC3143837

[39] Barkauskaite V Ek M Popovic K Harris HE Wahren-Herlenius M Nyberg F. Translocation of the novel cytokine HMGB1 to the cytoplasm and extracellular space coincides with the peak of clinical activity in experimentally UV-induced lesions of cutaneous lupus erythematosus. Lupus. (2007) 16:794–802. 10.1177/0961203307081895 17895302

[40] Verdelli A Coi A Marzano AV Antiga E Cozzani E Quaglino P. Autoantibody profile and clinical patterns in 619 Italian patients with cutaneous lupus erythematosus. J Eur Acad Dermatol Venereol. (2019) 33:742–52. 10.1111/jdv.15147 29924416

[41] Garcia S Campos-de-Carvalho AC. Neonatal lupus syndrome: the heart as a target of the immune system. An Acad Bras Cienc. (2000) 72:83–9. 10.1590/S0001-37652000000100012 10932109

[42] Hornberger LK Al Rajaa N. Spectrum of cardiac involvement in neonatal lupus. Scand J Immunol. (2010) 72:189–97. 10.1111/j.1365-3083.2010.02437.x 20696015

[43] Hon KL Leung AKC. Neonatal lupus erythematosus. Autoimmune Dis. (2012) 2012:301274. 10.1155/2012/301274 22973504 PMC3437607

[44] Barsalou J Costedoat-Chalumeau N Berhanu A Fors-Nieves C Shah U Brown P. Effect of in utero hydroxychloroquine exposure on the development of cutaneous neonatal lupus erythematosus. Ann Rheum Dis. (2018) 77:1742–9. 10.1136/annrheumdis-2018-213718 30297329 PMC6382275

[45] Shahian M Khosravi A Anbardar M-H. Early cholestasis in neonatal lupus erythematosus. Ann Saudi Med. (2011) 31:80–2. 10.5144/0256-4947.2011.80 20864789 PMC3101731

[46] Eftekhari P Sallé L Lezoualc'h F Mialet J Gastineau M Briand JP. Anti-SSA/Ro52 autoantibodies blocking the cardiac 5-HT4 serotoninergic receptor could explain neonatal lupus congenital heart block. Eur J Immunol. (2000) 30:2782–90. 10.1002/1521-4141(200010)30:10<2782::AID-IMMU2782>3.0.CO;2-9 11069058

[47] Buyon JP. Neonatal lupus: bedside to bench and back. Scand J Rheumatol. (1996) 25:271–6. 10.3109/03009749609104057 8921918

[48] Jaeggi E Laskin C Hamilton R Kingdom J Silverman E. The importance of the level of maternal anti-Ro/SSA antibodies as a prognostic marker of the development of cardiac neonatal lupus erythematosus a prospective study of 186 antibody-exposed fetuses and infants. J Am Coll Cardiol. (2010) 55:2778–84. 10.1016/j.jacc.2010.02.042 20538173

[49] Levy R Briggs L Silverman E Pope E Lara-Corrales I. Cutaneous sequelae in neonatal lupus: a retrospective cohort study. J Am Acad Dermatol. (2019). 10.1016/j.jaad.2019.09.083. [Epub ahead of print]. 31626881

[50] Hersh AO Arkin LM Prahalad S. Immunogenetics of cutaneous lupus erythematosus. Curr Opin Pediatr. (2016) 28:470–5. 10.1097/MOP.0000000000000383 27386968 PMC4962329

[51] Järvinen TM Hellquist A Koskenmies S Einarsdottir E Koskinen LLE Jeskanen L. Tyrosine kinase 2 and interferon regulatory factor 5 polymorphisms are associated with discoid and subacute cutaneous lupus erythematosus. Exp Dermatol. (2010) 19:123–31. 10.1111/j.1600-0625.2009.00982.x 19758313

[52] Fiehn C. Familial chilblain lupus - what can we learn from type I interferonopathies? Curr Rheumatol Rep. (2017) 19:61. 10.1007/s11926-017-0689-x 28844088

[53] König N Fiehn C Wolf C Schuster M Costa EC Tüngler V. Familial chilblain lupus due to a gain-of-function mutation in STING. Ann Rheum Dis. (2017) 76:468–472. 10.1136/annrheumdis-2016-209841 27566796

[54] Rice G Newman WG Dean J Patrick T Parmar R Flintoff K. Heterozygous mutations in TREX1 cause familial chilblain lupus and dominant aicardi-goutières syndrome. Am J Hum Genet. (2007) 80:811–5. 10.1086/513443 17357087 PMC1852703

[55] Zimmermann N Wolf C Schwenke R Lüth A Schmidt F Engel K. Assessment of clinical response to janus kinase inhibition in patients with familial chilblain lupus and TREX1 mutation. JAMA Dermatol. (2019) 155:342–6. 10.1001/jamadermatol.2018.5077 30673078 PMC6440279

[56] Günther C Berndt N Wolf C Lee-Kirsch MA. Familial chilblain lupus due to a novel mutation in the exonuclease III domain of 3' repair exonuclease 1 (TREX1). JAMA Dermatol. (2015) 151:426–31. 10.1001/jamadermatol.2014.3438 25517357

[57] Kuhn A Wenzel J Weyd H. Photosensitivity, apoptosis, and cytokines in the pathogenesis of lupus erythematosus: a critical review. Clin Rev Allergy Immunol. (2014) 47:148–62. 10.1007/s12016-013-8403-x 24420508

[58] Meller S Winterberg F Gilliet M Müller A Lauceviciute I Rieker J. Ultraviolet radiation–induced injury, chemokines, and leukocyte recruitment: an amplification cycle triggering cutaneous lupus erythematosus. Arthritis Rheum. (2005) 52:1504–16. 10.1002/art.21034 15880822

[59] Kochevar IE. Action spectrum and mechanisms of UV radiation-induced injury in lupus erythematosus. J Invest Dermatol. (1985) 85:S140–S3. 10.1111/1523-1747.ep12275658 2409182

[60] Furukawa F Itoh T Wakita H Yagi H Tokura Y Norris DA. Keratinocytes from patients with lupus erythematosus show enhanced cytotoxicity to ultraviolet radiation and to antibody-mediated cytotoxicity. Clin Exp Immunol. (1999) 118:164–70. 10.1046/j.1365-2249.1999.01026.x 10540174 PMC1905409

[61] Kuhn A Herrmann M Kleber S Beckmann-Welle M Fehsel K Martin-Villalba A. Accumulation of apoptotic cells in the epidermis of patients with cutaneous lupus erythematosus after ultraviolet irradiation. Arthritis Rheum. (2006) 54:939–50. 10.1002/art.21658 16511837

[62] Reefman E de Jong MC Kuiper H Jonkman MF Limburg PC Kallenberg CGM. Is disturbed clearance of apoptotic keratinocytes responsible for UVB-induced inflammatory skin lesions in systemic lupus erythematosus? Arthritis Res Ther. (2006) 8:R156. 10.1186/ar2051 17014704 PMC1794497

[63] Wolf SJ Estadt SN Gudjonsson JE Kahlenberg JM. Human and murine evidence for mechanisms driving autoimmune photosensitivity. Front Immunol. (2018) 9:2430. 10.3389/fimmu.2018.02430 30405625 PMC6205973

[64] Laurinaviciene R Sandholdt LH Bygum A. Drug-induced cutaneous lupus erythematosus: 88 new cases. Eur J Dermatol. (2017) 27:28–33. 10.1684/ejd.2016.2912 27799135

[65] Sandholdt LH Laurinaviciene R Bygum A. Proton pump inhibitor-induced subacute cutaneous lupus erythematosus. Br J Dermatol. (2014) 170:342–51. 10.1111/bjd.12699 24547721 PMC4232902

[66] Borucki R Werth VP. Cutaneous lupus erythematosus induced by drugs - novel insights. Expert Rev Clin Pharmacol. (2019) 13:35–42. 10.1080/17512433.2020.1698290 31774327

[67] Lowe GC Henderson CL Grau RH Hansen CB Sontheimer RD. A systematic review of drug-induced subacute cutaneous lupus erythematosus. Br J Dermatol. (2011) 164:465–72. 10.1111/j.1365-2133.2010.10110.x 21039412

[68] Reed BR Huff JC Jones SK Orton PW Lee LA Norris DA. Subacute cutaneous lupus erythematosus associated with hydrochlorothiazide therapy. Ann Intern Med. (1985) 103:49–51. 10.7326/0003-4819-103-1-49 3873891

[69] Kluger N Bessis D Guillot B. Chronic cutaneous lupus flare induced by systemic 5-fluorouracil. J Dermatolog Treat. (2006) 17:51–3. 10.1080/09546630500475591 16467025

[70] Weger W Kränke B Gerger A Salmhofer W Aberer E. Occurrence of subacute cutaneous lupus erythematosus after treatment with fluorouracil and capecitabine. J Am Acad Dermatol. (2008) 59:S4–S6. 10.1016/j.jaad.2007.06.040 18625380

[71] Aggarwal N. Drug-induced subacute cutaneous lupus erythematosus associated with proton pump inhibitors. Drugs Real World Outcomes. (2016) 3:145–54. 10.1007/s40801-016-0067-4 27398293 PMC4914530

[72] Reich A Maj J. Subacute cutaneous lupus erythematosus due to proton pump inhibitor intake: case report and literature review. Arch Med Sci. (2012) 8:743–7. 10.5114/aoms.2012.30300 23056090 PMC3460513

[73] Vedove CD Simon JC Girolomoni G. Drug-induced lupus erythematosus with emphasis on skin manifestations and the role of anti-TNFα agents. J Dtsch Dermatol Ges. (2012) 10:889–97. 10.1111/j.1610-0387.2012.08000.x 22937775 PMC3561694

[74] Boeckler P Cosnes A Francès C Hedelin G Lipsker D. Association of cigarette smoking but not alcohol consumption with cutaneous lupus erythematosus. Arch Dermatol. (2009) 145:1012–6. 10.1001/archdermatol.2009.199 19770440

[75] Miot HA Bartoli Miot LD Haddad GR. Association between discoid lupus erythematosus and cigarette smoking. Dermatology. (2005) 211:118–22. 10.1159/000086440 16088157

[76] Rahman P Gladman DD Urowitz MB. Smoking interferes with efficacy of antimalarial therapy in cutaneous lupus. J Rheumatol. (1998) 25:1716–9. 9733451

[77] Chasset F Francès C Barete S Amoura Z Arnaud L. Influence of smoking on the efficacy of antimalarials in cutaneous lupus: a meta-analysis of the literature. J Am Acad Dermatol. (2015) 72:634–9. 10.1016/j.jaad.2014.12.025 25648824

[78] Wattiaux A Bettendorf B Block L Gilmore-Bykovskyi A Ramly E Piper ME. Patient perspectives on smoking cessation and interventions in rheumatology clinics. Arthritis Care Res. (2019) 72:369–77. 10.1002/acr.23858 30768768 PMC6697238

[79] Cheng HM Liu WC Chua G Liew CF Li W Choo W. Impact of a pharmacy-led smoking cessation clinic in a dermatology centre. Singapore Med J. (2019) 60:31–3. 10.11622/smedj.2018063 29774362 PMC6351693

[80] Naranjo A Khan NA Cutolo M Lee S-S Lazovskis J Laas K. Smoking cessation advice by rheumatologists: results of an international survey. Rheumatology. (2014) 53:1825–9. 10.1093/rheumatology/keu213 24840678

[81] Masson C Audran M Pascaretti C Chevailler A Subra JF Tuchais E. Silica-associated systemic erythematosus lupus or mineral dust lupus? Lupus. (1997) 6:1–3. 10.1177/096120339700600101 9116713

[82] Vukicevic JS Milobratovic DJ. Discoid lupus erythematosus of the eyelid. Indian J Dermatol Venereol Leprol. (2010) 76:418–20. 10.4103/0378-6323.66592 20657131

[83] Sirobhushanam S Parsa N Reed TJ Berthier CC Sarkar MK Hile GA. *Staphylococcus aureus* colonization is increased on lupus skin lesions and is promoted by interferon-mediated barrier disruption. J Invest Dermatol. (2019) 140:1066–74.e4. 10.1016/j.jid.2019.11.016 31877319 PMC7183889

[84] Wenzel J. Cutaneous lupus erythematosus: new insights into pathogenesis and therapeutic strategies. Nat Rev Rheumatol. (2019) 15:519–32. 10.1038/s41584-019-0272-0 31399711

[85] Nagao K Kobayashi T Moro K Ohyama M Adachi T Kitashima DY. Stress-induced production of chemokines by hair follicles regulates the trafficking of dendritic cells in skin. Nat Immunol. (2012) 13:744–52. 10.1038/ni.2353 22729248 PMC4115277

[86] Shipman WD Chyou S Ramanathan A Izmirly PM Sharma S Pannellini T. A protective Langerhans cell-keratinocyte axis that is dysfunctional in photosensitivity. Sci Transl Med. (2018) 10:eaap9527. 10.1126/scitranslmed.aap9527 30111646 PMC6365282

[87] Hatakeyama M Fukunaga A Washio K Taguchi K Oda Y Ogura K. Anti-inflammatory role of langerhans cells and apoptotic keratinocytes in ultraviolet-B-induced cutaneous inflammation. J Immunol. (2017) 199:2937–47. 10.4049/jimmunol.1601681 28893957

[88] Sontheimer RD Bergstresser PR. Epidermal Langerhans cell involvement in cutaneous lupus erythematosus. J Invest Dermatol. (1982) 79:237–43. 10.1111/1523-1747.ep12500069 6215451

[89] Mori M Pimpinelli N Romagnoli P Bernacchi E Fabbri P Giannotti B. Dendritic cells in cutaneous lupus erythematosus: a clue to the pathogenesis of lesions. Histopathology. (1994) 24:311–21. 10.1111/j.1365-2559.1994.tb00531.x 8045520

[90] Berthier CC Tsoi LC Reed TJ Stannard JN Myers EM Namas R. Molecular profiling of cutaneous lupus lesions identifies subgroups distinct from clinical phenotypes. J Clin Med Res. (2019) 8:1244. 10.3390/jcm8081244 31426521 PMC6723404

[91] McNiff JM Kaplan DH. Plasmacytoid dendritic cells are present in cutaneous dermatomyositis lesions in a pattern distinct from lupus erythematosus. J Cutan Pathol. (2008) 35:452–6. 10.1111/j.1600-0560.2007.00848.x 18005168

[92] Farkas L Beiske K Lund-Johansen F Brandtzaeg P Jahnsen FL. Plasmacytoid dendritic cells (natural interferon-α/β-producing cells) accumulate in cutaneous lupus erythematosus lesions. Am J Pathol. (2001) 159:237–43. 10.1016/S0002-9440(10)61689-6 11438470 PMC1850412

[93] Tomasini D Mentzel T Hantschke M Cerri A Paredes B Rütten A. Plasmacytoid dendritic cells: an overview of their presence and distribution in different inflammatory skin diseases, with special emphasis on Jessner's lymphocytic infiltrate of the skin and cutaneous lupus erythematosus. J Cutan Pathol. (2010) 37:1132–9. 10.1111/j.1600-0560.2010.01587.x 20659210

[94] Yin Q Xu X Lin Y Lv J Zhao L He R. Ultraviolet B irradiation induces skin accumulation of plasmacytoid dendritic cells: a possible role for chemerin. Autoimmunity. (2014) 47:185–92. 10.3109/08916934.2013.866105 24328602

[95] Furie R Werth VP Merola JF Stevenson L Reynolds TL Naik H. Monoclonal antibody targeting BDCA2 ameliorates skin lesions in systemic lupus erythematosus. J Clin Invest. (2019) 129:1359–71. 10.1172/JCI124466 30645203 PMC6391094

[96] Safi R Al-Hage J Abbas O Kibbi A-G Nassar D. Investigating the presence of neutrophil extracellular traps in cutaneous lesions of different subtypes of lupus erythematosus. Exp Dermatol. (2019) 28:1348–52. 10.1111/exd.14040 31529548

[97] Villanueva E Yalavarthi S Berthier CC Hodgin JB Khandpur R Lin AM. Netting neutrophils induce endothelial damage, infiltrate tissues, and expose immunostimulatory molecules in systemic lupus erythematosus. J Immunol. (2011) 187:538–52. 10.4049/jimmunol.1100450 21613614 PMC3119769

[98] Guo X Fang X He G Zaman MH Fei X Qiao W. The role of neutrophils in skin damage induced by tissue-deposited lupus IgG. Immunology. (2018) 154:604–12. 10.1111/imm.12908 29450882 PMC6050218

[99] Nakajima M Nakajima A Kayagaki N Honda M Yagita H Okumura K. Expression of Fas ligand and its receptor in cutaneous lupus: implication in tissue injury. Clin Immunol Immunopathol. (1997) 83:223–9. 10.1006/clin.1997.4352 9175910

[100] Liu J Berthier CC Kahlenberg JM. Enhanced inflammasome activity in systemic lupus erythematosus is mediated via type I interferon upregulation of interferon regulatory factor 1. Arthritis Rheumatol. (2017) 69:1840–9. 10.1002/art.40166 28564495 PMC5575977

[101] Masek-Hammerman K Peeva E Ahmad A Menon S Afsharvand M Peng Qu R. Monoclonal antibody against macrophage colony-stimulating factor suppresses circulating monocytes and tissue macrophage function but does not alter cell infiltration/activation in cutaneous lesions or clinical outcomes in patients with cutaneous lupus erythematosus. Clin Exp Immunol. (2016) 183:258–70. 10.1111/cei.12705 26376111 PMC4711167

[102] Korb LC Ahearn JM. C1q binds directly and specifically to surface blebs of apoptotic human keratinocytes: complement deficiency and systemic lupus erythematosus revisited. J Immunol. (1997) 158:4525–8. 9144462

[103] Racila DM Sontheimer CJ Sheffield A Wisnieski JJ Racila E Sontheimer RD. Homozygous single nucleotide polymorphism of the complement C1QA gene is associated with decreased levels of C1q in patients with subacute cutaneous lupus erythematosus. Lupus. (2003) 12:124–32. 10.1191/0961203303lu329oa 12630757

[104] Wenzel J Zahn S Mikus S Wiechert A Bieber T Tüting T. The expression pattern of interferon-inducible proteins reflects the characteristic histological distribution of infiltrating immune cells in different cutaneous lupus erythematosus subsets. Br J Dermatol. (2007) 157:752–7. 10.1111/j.1365-2133.2007.08137.x 17714558

[105] Sinha AA Dey-Rao R. Genomic Investigation of Lupus in the Skin. J Investig Dermatol Symp Proc. (2017) 18:S75–S80. 10.1016/j.jisp.2016.09.002 28941499

[106] Lipsker D Saurat J-H. Neutrophilic cutaneous lupus erythematosus. Dermatology. (2008) 216:283. 10.1159/000113940 18230975

[107] Lu L Kaliyaperumal A Boumpas DT Datta SK. Major peptide autoepitopes for nucleosome-specific T cells of human lupus. J Clin Invest. (1999) 104:345–55. 10.1172/JCI6801 10430616 PMC408421

[108] Voll RE Roth EA Girkontaite I Fehr H Herrmann M Lorenz H-M. Histone-specific Th0 and Th1 clones derived from systemic lupus erythematosus patients induce double-stranded DNA antibody production. Arthritis Rheum. (1997) 40:2162–71. 10.1002/art.1780401210 9416853

[109] Furukawa F Tokura Y Matsushita K Iwasaki-Inuzuka K Onagi-Suzuki K Yagi H. Selective expansions of T cells expressing Vβ38 and Vβ13 in skin lesions of patients with chronic cutaneous lupus erythematosus. J Dermatol. (1996) 23:670–6. 10.1111/j.1346-8138.1996.tb02679.x 8973031

[110] Wenzel J Zahn S Bieber T Tüting T. Type I interferon-associated cytotoxic inflammation in cutaneous lupus erythematosus. Arch Dermatol Res. (2009) 301:83–6. 10.1007/s00403-008-0892-8 18784932

[111] Grassi M Capello F Bertolino L Seia Z. Identification of granzyme B-expressing CD-8-positive T cells in lymphocytic inflammatory infiltrate in cutaneous lupus erythematosus and in dermatomyositis. Clin Exp Dermatol. (2009) 34:910–4. 10.1111/j.1365-2230.2009.03297.x 19456762

[112] Luo Y Zhang X Zhao M Lu Q. DNA demethylation of the perforin promoter in CD4^+^ T cells from patients with subacute cutaneous lupus erythematosus. J Dermatol Sci. (2009) 56:33–6. 10.1016/j.jdermsci.2009.06.010 19651491

[113] Luo Y Zhao M Lu Q. Demethylation of promoter regulatory elements contributes to CD70 overexpression in CD4^+^ T cells from patients with subacute cutaneous lupus erythematosus. Clin Exp Dermatol. (2010) 35:425–30. 10.1111/j.1365-2230.2009.03611.x 19874375

[114] Luo Y Li Y Su Y Yin H Hu N Wang S. Abnormal DNA methylation in T cells from patients with subacute cutaneous lupus erythematosus. Br J Dermatol. (2008) 159:827–33. 10.1111/j.1365-2133.2008.08758.x 18644019

[115] Wenzel J Henze S Brähler S Bieber T Tüting T. The expression of human leukocyte antigen-DR and CD25 on circulating T cells in cutaneous lupus erythematosus and correlation with disease activity. Exp Dermatol. (2005) 14:454–9. 10.1111/j.0906-6705.2005.00301.x 15885081

[116] Franz B Fritzsching B Riehl A Oberle N Klemke C-D Sykora J. Low number of regulatory T cells in skin lesions of patients with cutaneous lupus erythematosus. Arthritis Rheum. (2007) 56:1910–20. 10.1002/art.22699 17530636

[117] Gambichler T Pätzholz J Schmitz L Lahner N Kreuter A. FOXP3^+^ and CD39^+^ regulatory T cells in subtypes of cutaneous lupus erythematosus. J Eur Acad Dermatol Venereol. (2015) 29:1972–7. 10.1111/jdv.13123 25808110

[118] Volc-Platzer B Anegg B Milota S Pickl W Fischer G. Accumulation of γ*δ* cells in chronic cutaneous lupus erythematosus. J Invest Dermatol. (1993) 100:S84–S91. 10.1038/jid.1993.29 8423404

[119] Walsh NM Kutzner H Requena L Cerroni L. Plasmacytic cutaneous pathology: a review. J Cutan Pathol. (2019) 46:698–708. 10.1111/cup.13499 31095757

[120] Chan O Shlomchik MJ. A new role for B cells in systemic autoimmunity: B cells promote spontaneous T cell activation in MRL-lpr/lpr mice. J Immunol. (1998) 160:51–9. 9551955

[121] Shlomchik MJ Madaio MP Ni D Trounstein M Huszar D. The role of B cells in lpr/lpr-induced autoimmunity. J Exp Med. (1994) 180:1295–306. 10.1084/jem.180.4.1295 7931063 PMC2191708

[122] Chan OT Hannum LG Haberman AM Madaio MP Shlomchik MJ. A novel mouse with B cells but lacking serum antibody reveals an antibody-independent role for B cells in murine lupus. J Exp Med. (1999) 189:1639–48. 10.1084/jem.189.10.1639 10330443 PMC2193634

[123] Kansal R Richardson N Neeli I Khawaja S Chamberlain D Ghani M. Sustained B cell depletion by CD19-targeted CAR T cells is a highly effective treatment for murine lupus. Sci Transl Med. (2019) 11:eaav1648. 10.1126/scitranslmed.aav1648 30842314 PMC8201923

[124] Correll CK Miller DD Maguiness SM. Treatment of childhood-onset lupus erythematosus panniculitis with rituximab. JAMA Dermatol. (2020) 156:566–9. 10.1001/jamadermatol.2019.4984 32049306 PMC7042827

[125] Hofmann SC Leandro MJ Morris SD Isenberg DA. Effects of rituximab-based B-cell depletion therapy on skin manifestations of lupus erythematosus–report of 17 cases and review of the literature. Lupus. (2013) 22:932–9. 10.1177/0961203313497115 23894047 PMC4107853

[126] Herati RS Muselman A Vella L Bengsch B Parkhouse K Del Alcazar D. Successive annual influenza vaccination induces a recurrent oligoclonotypic memory response in circulating T follicular helper cells. Sci Immunol. (2017) 2:eaag2152 10.1126/sciimmunol.aag2152 28620653 PMC5469419

[127] González-Escribano MF Aguilar F Torres B Sánchez-Román J Núñez-Roldán A. CD38 polymorphisms in Spanish patients with systemic lupus erythematosus. Hum Immunol. (2004) 65:660–4. 10.1016/j.humimm.2004.02.032 15219386

[128] Viegas MS Silva T Monteiro MM do Carmo A Martins TC. Knocking out of CD38 accelerates development of a lupus-like disease in lpr mice. Rheumatology. (2011) 50:1569–77. 10.1093/rheumatology/ker178 21586522

[129] Erkeller-Yuksel FM Lydyard PM Isenberg DA. Lack of NK cells in lupus patients with renal involvement. Lupus. (1997) 6:708–12. 10.1177/096120339700600905 9412985

[130] Green MRJ Kennell ASM Larche MJ Seifert MH Isenberg DA Salaman MR. Natural killer cell activity in families of patients with systemic lupus erythematosus: demonstration of a killing defect in patients. Clin Exp Immunol. (2005) 141:165–73. 10.1111/j.1365-2249.2005.02822.x 15958083 PMC1809425

[131] Henriques A Teixeira L Inês L Carvalheiro T Gonçalves A Martinho A. NK cells dysfunction in systemic lupus erythematosus: relation to disease activity. Clin Rheumatol. (2013) 32:805–13. 10.1007/s10067-013-2176-8 23377197

[132] Park Y-W Kee S-J Cho Y-N Lee E-H Lee H-Y Kim E-M. Impaired differentiation and cytotoxicity of natural killer cells in systemic lupus erythematosus. Arthritis Rheum. (2009) 60:1753–63. 10.1002/art.24556 19479851

[133] Schepis D Gunnarsson I Eloranta M-L Lampa J Jacobson SH Kärre K. Increased proportion of CD56bright natural killer cells in active and inactive systemic lupus erythematosus. Immunology. (2009) 126:140–6. 10.1111/j.1365-2567.2008.02887.x 18564343 PMC2632704

[134] Huang Z Fu B Zheng SG Li X Sun R Tian Z. Involvement of CD226^+^ NK cells in immunopathogenesis of systemic lupus erythematosus. J Immunol. (2011) 186:3421–31. 10.4049/jimmunol.1000569 21296979 PMC3097030

[135] Hervier B Beziat V Haroche J Mathian A. Phenotype and function of natural killer cells in systemic lupus erythematosus: excess interferon-γ production in patients with active disease. Arthritis. (2011) 63:1698–706. 10.1002/art.30313 21370226

[136] Raulet DH Guerra N. Oncogenic stress sensed by the immune system: role of natural killer cell receptors. Nat Rev Immunol. (2009) 9:568–80. 10.1038/nri2604 19629084 PMC3017432

[137] Swann JB Hayakawa Y Zerafa N Sheehan KCF Scott B Schreiber RD. Type I IFN contributes to NK cell homeostasis, activation, and antitumor function. J Immunol. (2007) 178:7540–9. 10.4049/jimmunol.178.12.7540 17548588

[138] Smyth MJ Hayakawa Y Takeda K Yagita H. New aspects of natural-killer-cell surveillance and therapy of cancer. Nat Rev Cancer. (2002) 2:850–61. 10.1038/nrc928 12415255

[139] Sullivan TP Dearaujo T Vincek V Berman B. Evaluation of superficial basal cell carcinomas after treatment with imiquimod 5% cream or vehicle for apoptosis and lymphocyte phenotyping. Dermatol Surg. (2003) 29:1181–6. 10.1097/00042728-200312000-00007 14725659

[140] Nitahara A Shimura H Ito A Tomiyama K Ito M Kawai K. NKG2D ligation without T cell receptor engagement triggers both cytotoxicity and cytokine production in dendritic epidermal T cells. J Invest Dermatol. (2006) 126:1052–8. 10.1038/sj.jid.5700112 16484989

[141] Hofmann SC Bosma A Bruckner-Tuderman L Vukmanovic-Stejic M Jury EC Isenberg DA. Invariant natural killer T cells are enriched at the site of cutaneous inflammation in lupus erythematosus. J Dermatol Sci. (2013) 71:22–8. 10.1016/j.jdermsci.2013.04.012 23664188

[142] Wenzel J Wörenkämper E Freutel S Henze S Haller O Bieber T. Enhanced type I interferon signalling promotes Th1-biased inflammation in cutaneous lupus erythematosus. J Pathol. (2005) 205:435–42. 10.1002/path.1721 15685590

[143] Flier J Boorsma DM van Beek PJ Nieboer C Stoof TJ Willemze R. Differential expression of CXCR3 targeting chemokines CXCL10, CXCL9, and CXCL11 in different types of skin inflammation. J Pathol. (2001) 194:398–405. 10.1002/1096-9896(200108)194:4<397::AID-PATH899>3.0.CO;2-S 11523046

[144] Denton AE Innocentin S Carr EJ Bradford BM Lafouresse F Mabbott NA. Type I interferon induces CXCL13 to support ectopic germinal center formation. J Exp Med. (2019) 216:621–37. 10.1084/jem.20181216 30723095 PMC6400543

[145] Moser B. CXCR5, the defining marker for follicular B helper T (TFH) Cells. Front Immunol. (2015) 6:296. 10.3389/fimmu.2015.00296 26106395 PMC4459225

[146] Niederkorn A Frühauf J Schwantzer G Wutte N Painsi C Werner S. CXCL13 is an activity marker for systemic, but not cutaneous lupus erythematosus: a longitudinal cohort study. Arch Dermatol Res. (2018) 310:485–93. 10.1007/s00403-018-1836-6 29728857

[147] Clark KL Reed TJ Wolf SJ Lowe L Hodgin JB Kahlenberg JM. Epidermal injury promotes nephritis flare in lupus-prone mice. J Autoimmun. (2015) 65:38–48. 10.1016/j.jaut.2015.08.005 26305061 PMC4679658

[148] Sáez de Guinoa J Barrio L Mellado M Carrasco YR. CXCL13/CXCR5 signaling enhances BCR-triggered B-cell activation by shaping cell dynamics. Blood. (2011) 118:1560–9. 10.1182/blood-2011-01-332106 21659539

[149] Wenzel J Henze S Wörenkämper E Basner-Tschakarjan E Sokolowska-Wojdylo M Steitz J. Role of the chemokine receptor CCR4 and its ligand thymus- and activation-regulated chemokine/CCL17 for lymphocyte recruitment in cutaneous lupus erythematosus. J Invest Dermatol. (2005) 124:1241–8. 10.1111/j.0022-202X.2005.23755.x 15955100

[150] Freutel S Gaffal E Zahn S Bieber T Tüting T Wenzel J. Enhanced CCR5^+^/CCR3^+^ T helper cell ratio in patients with active cutaneous lupus erythematosus. Lupus. (2011) 20:1300–4. 10.1177/0961203311409267 21844117

[151] Mande P Zirak B Ko W-C Taravati K Bride KL Brodeur TY. Fas ligand promotes an inducible TLR-dependent model of cutaneous lupus-like inflammation. J Clin Invest. (2018) 128:2966–78. 10.1172/JCI98219 29889098 PMC6025993

[152] Merrill JT Furie R Werth VP Khamashta M Drappa J Wang L. Anifrolumab effects on rash and arthritis: impact of the type I interferon gene signature in the phase IIb MUSE study in patients with systemic lupus erythematosus. Lupus Sci Med. (2018) 5:e000284. 10.1136/lupus-2018-000284 30588322 PMC6280909

[153] Furie R Khamashta M Merrill JT Werth VP Kalunian K Brohawn P. Anifrolumab, an anti–interferon-α receptor monoclonal antibody, in moderate-to-severe systemic lupus erythematosus. Arthritis Rheumatol. (2017) 69:376–86. 10.1002/art.39962 28130918 PMC5299497

[154] Felten R Scher F Sagez F Chasset F Arnaud L. Spotlight on anifrolumab and its potential for the treatment of moderate-to-severe systemic lupus erythematosus: evidence to date. Drug Des Devel Ther. (2019) 13:1535–43. 10.2147/DDDT.S170969 31190735 PMC6514126

[155] Werth VP Fiorentino D Sullivan BA Boedigheimer MJ Chiu K Wang C. Brief report: pharmacodynamics, safety, and clinical efficacy of AMG 811, a human anti-interferon-γ antibody, in patients with discoid lupus erythematosus. Arthritis Rheumatol. (2017) 69:1028–34. 10.1002/art.40052 28118537 PMC5434930

[156] Tsoi LC Hile GA Berthier CC Sarkar MK Reed TJ Liu J. Hypersensitive IFN responses in lupus keratinocytes reveal key mechanistic determinants in cutaneous lupus. J Immunol. (2019) 202:2121–30. 10.4049/jimmunol.1800650 30745462 PMC6424612

[157] Sarkar MK Hile GA Tsoi LC Xing X Liu J Liang Y. Photosensitivity and type I IFN responses in cutaneous lupus are driven by epidermal-derived interferon kappa. Ann Rheum Dis. (2018) 77:1653–64. 10.1136/annrheumdis-2018-213197 30021804 PMC6185784

[158] Stannard JN Reed TJ Myers E Lowe L Sarkar MK Xing X. Lupus Skin is primed for IL-6 inflammatory responses through a keratinocyte-mediated autocrine type I interferon loop. J Invest Dermatol. (2017) 137:115–22. 10.1016/j.jid.2016.09.008 27646883 PMC5183476

[159] Salvi V Vermi W Cavani A Lonardi S Carbone T Facchetti F. IL-21 may promote granzyme B-dependent NK/plasmacytoid dendritic cell functional interaction in cutaneous lupus erythematosus. J Invest Dermatol. (2017) 137:1493–500. 10.1016/j.jid.2017.03.016 28344062

[160] Karrich JJ Jachimowski LCM Nagasawa M Kamp A Balzarolo M Wolkers MC. IL-21-stimulated human plasmacytoid dendritic cells secrete granzyme B, which impairs their capacity to induce T-cell proliferation. Blood. (2013) 121:3103–11. 10.1182/blood-2012-08-452995 23407551 PMC3630825

[161] Yell JA Burge SM. The effect of hormonal changes on cutaneous disease in lupus erythematosus. Br J Dermatol. (1993) 129:18–22. 10.1111/j.1365-2133.1993.tb03305.x 8369206

[162] Jones SK. The effects of hormonal and other stimuli on cell-surface Ro/SSA antigen expression by human keratinocytes in vitro: their possible role in the induction of cutaneous lupus lesions. Br J Dermatol. (1992) 126:554–60. 10.1111/j.1365-2133.1992.tb00099.x 1319192

[163] Furukawa F Lyons MB Lee LA. Estradiol enhances binding to cultured human keratinocytes of antibodies specific for SS-A/Ro and SS-B/La. Another possible mechanism for estradiol influence of Lupus Erythematosus. J Immunol. (1988) 141:1480–8. 2457617

[164] Zandman-Goddard G Solomon M Barzilai A Shoenfeld Y. Lupus erythematosus tumidus induced by sex reassignment surgery. J Rheumatol. (2007) 34:1938–40. 17696264

[165] Fox HS Bond BL Parslow TG. Estrogen regulates the IFN-gamma promoter. J Immunol. (1991) 146:4362–7. 1904081

[166] Sorachi K-I Kumagai S Sugita M Yodoi J Imura H. Enhancing effect of 17β-estradiol on human NK cell activity. Immunol Lett. (1993) 36:31–5. 10.1016/0165-2478(93)90065-A 8344714

[167] Liang Y Tsoi LC Xing X Beamer MA Swindell WR Sarkar MK. A gene network regulated by the transcription factor VGLL3 as a promoter of sex-biased autoimmune diseases. Nat Immunol. (2016) 18:152. 10.1038/ni.3643 27992404 PMC5289297

[168] Billi AC Gharaee-Kermani M Fullmer J Tsoi LC Hill BD Gruszka D. The female-biased factor VGLL3 drives cutaneous and systemic autoimmunity. JCI Insight. (2019) 4:e127291. 10.1172/jci.insight.127291 30996136 PMC6538382

[169] Klaeschen AS Wolf D Brossart P Bieber T Wenzel J. JAK inhibitor ruxolitinib inhibits the expression of cytokines characteristic of cutaneous lupus erythematosus. Exp Dermatol. (2017) 26:728–30. 10.1111/exd.13253 27892610

[170] Dörner T Tanaka Y Petri M Smolen JS Dow ER Higgs RE Benschop RJ Abel A Silk ME de Bono S 185 Baricitinib-associated changes in type I interferon gene signature during a 24-week phase 2 clinical SLE trial. Lupus Sci Med. (2019) 6:A141 10.1136/lupus-2019-lsm.185 PMC7549481 33037080

[171] Wallace DJ Furie RA Tanaka Y Kalunian KC Mosca M Petri MA. Baricitinib for systemic lupus erythematosus: a double-blind, randomised, placebo-controlled, phase 2 trial. Lancet. (2018) 392:222–31. 10.1016/S0140-6736(18)31363-1 30043749

[172] Namour F Desrivot J Van der Aa A Harrison P Tasset C van't Klooster G. Clinical confirmation that the selective JAK1 inhibitor filgotinib (GLPG0634) has a low liability for drug-drug interactions. Drug Metab Lett. (2016) 10:38–48. 10.2174/1872312810666151223103353 26693854

[173] Baker KF Isaacs JD. Novel therapies for immune-mediated inflammatory diseases: what can we learn from their use in rheumatoid arthritis, spondyloarthritis, systemic lupus erythematosus, psoriasis, Crohn's disease and ulcerative colitis? Ann Rheum Dis. (2018) 77:175–87. 10.1136/annrheumdis-2017-211555 28765121

[174] Pohlmeyer C Cui Z-H Han P Clarke AS Jones RM Mollova N AB0484 Monotherapy with filgotinib, a jak1-selective inhibitor, reduces disease severity and alters immune cell subsets in the nzb/w f1 murine model of lupus. Ann Rheum Dis. (2018) 77:1403 10.1136/annrheumdis-2018-eular.3367

[175] Van Vollenhoven RF Hahn BH Tsokos GC Wagner CL Lipsky P Touma Z. Efficacy and safety of ustekinumab, an IL-12 and IL-23 inhibitor, in patients with active systemic lupus erythematosus: results of a multicentre, double-blind, phase 2, randomised, controlled study. Lancet. (2018) 392:1330–9. 10.1016/S0140-6736(18)32167-6 30249507

[176] Romero-Maté A García-Donoso C Hernández-Núñez A Martínez-Morán C Moreno-Torres A Borbujo-Martínez J. Successful treatment of recalcitrant discoid lupus erythematosus with ustekinumab. Dermatol Online J. (2017) 23:13030/qt206538zm. 28329473

[177] Dahl C Johansen C Kragballe K Olesen AB. Ustekinumab in the treatment of refractory chronic cutaneous lupus erythematosus: a case report. Acta Derm Venereol. (2013) 93:368–9. 10.2340/00015555-1467 23038045

[178] De Souza A Ali-Shaw T Strober BE Franks AG Jr. Successful treatment of subacute lupus erythematosus with ustekinumab. Arch Dermatol. (2011) 147:896–8. 10.1001/archdermatol.2011.185 21844448

[179] Tierney E Kirthi S Ramsay B Ahmad K. Ustekinumab-induced subacute cutaneous lupus. JAAD Case Rep. (2019) 5:271–3. 10.1016/j.jdcr.2019.01.015 30891478 PMC6403108

[180] Urowitz MB Isenberg DA Wallace DJ. Safety and efficacy of hCDR1 (Edratide) in patients with active systemic lupus erythematosus: results of phase II study. Lupus Sci Med. (2015) 2:e000104. 10.1136/lupus-2015-000104 26301100 PMC4538379

[181] Skuljec J Chmielewski M Happle C Habener A Busse M Abken H. Chimeric antigen receptor-redirected regulatory T cells suppress experimental allergic airway inflammation, a model of asthma. Front Immunol. (2017) 8:1125. 10.3389/fimmu.2017.01125 28955341 PMC5600908

[182] Mizui M Tsokos GC. Targeting regulatory T cells to treat patients with systemic lupus erythematosus. Front Immunol. (2018) 9:786. 10.3389/fimmu.2018.00786 29755456 PMC5932391

